# Behavioral Profiles and Sociodemographic Predictors of Planetary Health Diet Engagement Among Health Care Professionals to Inform Public Health Promotion: Cross-Sectional Study

**DOI:** 10.2196/67633

**Published:** 2025-11-12

**Authors:** Florence Carrouel, Rita Nugem, Laurie Fraticelli, Yohan Fayet, Lama Basbous, Corélie Salque, Claude Dussart, Marie-Thérèse Charreyre, Romain Lan, Audrey Murat-ringot

**Affiliations:** 1 Health Systemic Process Laboratory (P2S), UR4129 University Claude Bernard Lyon 1 University of Lyon Lyon France; 2 Department of Geography Université Clermont Auvergne, AgroParisTech, INRAE Clermont-Ferrand France; 3 Hospices Civils de Lyon Lyon France; 4 Universite Claude Bernard Lyon 1, INSA Lyon, Université Jean Monnet, Centre National de la Recherche Scientifique (CNRS) UMR 5223, Ingénierie des Matériaux Polymères Villeurbanne France; 5 Laboratory Anthropologie Bio-Culturelle, Droit, Éthique et Santé (ADES) Aix Marseille University, Centre National de la Recherche Scientifique (CNRS), Établissement Français du Sang (EFS) Marseille France

**Keywords:** planetary health diet, sustainable nutrition, food systems, dietary transition, One Health

## Abstract

**Background:**

The planetary health diet (PHD) promotes dietary habits that are beneficial to human health and environmental sustainability, two closely related goals of modern public health. Health care professionals are expected to lead by example and use their position to encourage their patients and communities to adopt healthier and more sustainable behaviors. However, little is known about health care professionals’ knowledge, attitudes, and behaviors (KAB) related to the PHD, which limits the ability to design targeted interventions for this key population.

**Objective:**

This study aimed to analyze KAB profiles related to the PHD among French health care professionals and assess associations with individual characteristics.

**Methods:**

This cross-sectional study was conducted from April 2024 to June 2024 among health care professionals. Participants were recruited using a nonprobabilistic convenience sampling method. Inclusion criteria were to be aged ≥18 years, fluent in French, and working as a health care professional at Hospices Civils de Lyon. Data were collected through an online questionnaire including sociodemographic and geographic variables, KAB items related to the PHD, and dietary intake. Adherence to the PHD was calculated using a validated scoring system. KAB items were analyzed using k-means clustering to identify distinct profiles. Associations with sociodemographic and geographic variables were explored using chi-square tests and ANOVA.

**Results:**

Among 1104 respondents (n=927, 83.97% women and n=882, 79.89% aged between 30 and 59 years), 3 KAB clusters were identified: cluster 1 (“knowledge-attitude gap”; n=481, 43.57%) included professionals with good knowledge but moderate attitudes and partially consistent behaviors; cluster 2 (“high intent, low action”; n=318, 28.8 included those with strong knowledge and positive attitudes but limited behavioral change, and cluster 3 (“behavior-driven alignment”; n=305, 27.63%) included participants who reported pro-PHD behaviors despite lower knowledge and less favorable attitudes. PHD adherence was significantly associated with being female, having a vegetarian or flexitarian diet, and reporting environmental concerns (*P*<.01 in all cases).

**Conclusions:**

Distinct KAB profiles among health care professionals suggest differing levels of engagement regarding the PHD. Findings suggest that tailored interventions addressing knowledge, attitudes, or behaviors could improve health care professionals’ alignment with planetary health goals within similar institutional contexts.

## Introduction

The planetary health diet (PHD), introduced by the EAT-Lancet Commission, represents a transformative dietary paradigm that seeks to simultaneously promote human health and environmental sustainability [1]. Since the publication of the EAT-Lancet report, growing empirical evidence has confirmed the dual benefit of planetary health–aligned diets for both human and environmental outcomes. Recent global analyses show that higher adherence to plant-forward dietary patterns reduces the incidence of noncommunicable diseases (NCDs) and mortality while simultaneously decreasing greenhouse gas emissions and land use [2-4]. In addition, moderate shifts toward sustainable diets could prevent millions of premature deaths annually and contribute significantly to climate mitigation targets [5,6]. Beyond individuals, health care systems themselves are increasingly recognized as strategic environments for implementing and modeling sustainable food choices [7,8]. Health care professionals are also increasingly viewed as key actors in the transition toward climate-resilient and sustainable health systems. Recent reports emphasize their dual role as credible messengers and institutional change agents in promoting planetary health principles within hospitals and clinical settings [9-11].

Poor dietary patterns are among the leading modifiable risk factors of NCDs, which account for approximately 74% of global deaths [12]. Diets high in ultraprocessed foods, red and processed meats, added sugars, and saturated fats have been strongly linked to cardiovascular disease, type 2 diabetes, obesity, certain cancers, and chronic respiratory diseases [13,14]. Moreover, many countries—particularly in rapidly urbanizing regions—face a double burden of malnutrition, where undernutrition and overweight or obesity coexist within the same communities or even individuals [15]. Nutrition is recognized as a key pathway through which social determinants of health exert their effects. Socioeconomic factors, education, and environmental contexts shape dietary behaviors and contribute to health disparities [16]. Consequently, transforming food systems is vital not only for curbing NCDs but also for securing long-term planetary health.

Responding to these interlinked crises, the field of planetary health has emerged to integrate human health with the sustainability of the Earth’s ecosystems [17]. The PHD provides a concrete application of this vision, aligning nutritional science with environmental goals. However, despite its potential, empirical studies suggest a persistent “knowledge-attitude gap”: although awareness of healthy and sustainable diets is growing, actual behavioral adoption remains limited [18]. This discrepancy highlights the complex interplay of psychological, social, and structural factors that shape dietary behavior.

To investigate this complexity, the knowledge, attitudes, and behaviors (KAB) model offers a useful lens. Rooted in health psychology, it posits that knowledge influences attitudes, which subsequently guide behaviors [19,20]. However, real-world deviations from this linear sequence suggest the need to explore individual variability in how people relate to new dietary models such as the PHD.

Cluster analysis has emerged as a powerful tool to address such variability by identifying subgroups within populations based on shared patterns of KAB [21]. Identifying such patterns is especially important among health care professionals, who are expected to champion evidence-based health behaviors and influence broader public adoption.

This study aimed to identify distinct profiles of health care professionals based on their KAB regarding the PHD and investigate which sociodemographic factors are associated with these behavioral profiles. By answering this research question, this study sought to contribute to the development of more tailored and effective strategies for promoting sustainable dietary transitions in line with the goals of human and planetary health.

## Methods

### Study Design

This study was a descriptive, cross-sectional, and quantitative approach to examine KAB related to the PHD adhering to the Checklist for Reporting of Survey Studies (Multimedia Appendix 1). It constitutes the behavioral component of the BIOQUALIM project [22], a transdisciplinary One Health research program assessing the role of the PHD in the primary and tertiary prevention of NCDs through 4 complementary studies (clinical, chemical, behavioral, and psychosocial).

### Study Setting

This study was conducted in France at the Hospices Civils de Lyon (HCL) between April 15, 2024, and June 15, 2024. HCL is the second-largest public university hospital network in France, encompassing 13 hospitals and approximately 24,000 employees across the Lyon metropolitan area [23]. It covers a wide range of clinical specialties, research units, and training departments. The workforce includes physicians, nurses, midwives, pharmacists, technicians, allied health staff, and administrative personnel, constituting a highly diverse and representative health care environment.

### Participant Eligibility

The participants included (1) were aged ≥18 years, (2) spoke fluent French, and (3) were health care professionals at HCL.

### Recruitment and Data Collection Procedures

#### Sampling Strategy

A nonprobabilistic convenience sampling method was used. A systematic dissemination plan was implemented across all HCL hospital sites to ensure broad and consistent recruitment coverage. The survey was distributed in both digital and paper formats. Digital invitations were circulated via institutional mailing lists targeting all professional categories within the HCL system. A pop-up link was displayed on the hospital intranet and on shared computers in staff rooms and administrative offices. Flyers with QR codes were also distributed on meal trays and posted in common areas. This multichannel strategy ensured that both clinical and nonclinical staff could access the survey regardless of their work schedule or digital access constraints.

#### Questionnaire Development and Validation

The survey was created to assess KAB regarding the PHD and was based on a literature review and the World Health Organization’s (WHO) methodology for designing KAB surveys [24]. Initial items were inspired by established tools, notably the Food Choice Questionnaire [25]. After generating a preliminary version of 136 closed questions, expert review was undertaken to validate both face and content aspects. An expert panel—including nutritionists, dietitians, and general practitioners—reviewed and refined the items. Cognitive testing was conducted through qualitative cognitive interviews with 20 volunteer respondents using think-aloud and probing techniques to evaluate comprehension and clarity of the items in line with best practices for questionnaire pretesting [26]. The final validated version comprised 88 scored items across knowledge (n=36), attitudes (n=21), and behavior (n=31) domains, plus 1 open-ended question [22].

#### Scoring System

To quantify responses, a scoring system was developed through a Delphi consensus process involving a panel of experts. Knowledge items were assessed dichotomously (+1 for correct and −1 for incorrect answers) as they referred to factual information with a single correct answer.

In contrast, attitude and behavior items were initially designed using Likert-type scales (eg, frequency or level of agreement). To ensure comparability and interpretive coherence across the 3 KAB dimensions, these Likert-type responses were subsequently transformed into normalized values (+1, 0, and −1) by the expert panel. This transformation reflected the degree of alignment between each response and the principles of the PHD as determined through consensus among Delphi participants [24,27,28].

This standardized conversion was chosen to maintain the semantic richness of the original Likert items while allowing for a unified scoring framework across domains. It also minimized subjective interpretation and facilitated statistical aggregation of KAB scores within cluster analysis. Such normalization is consistent with WHO knowledge, attitude, and practice methodologies, which favor simplified, direction-oriented coding to describe behavioral tendencies at the population level [24,29].

Scores for each KAB dimension were normalized on a scale from 0 to 40, with total KAB scores ranging from 0 to 120. Categories were set as follows: inadequate (0-70), marginal (71-88), and adequate (89-120).

#### Survey Platform

Participants completed the questionnaire using LimeSurvey (version 6.5.7; LimeSurvey GmbH). Measures were taken to ensure secure, anonymous participation: CAPTCHA verification, cookies to block duplicate submissions, and unique tokens for log-in. Respondents could pause and resume the survey as needed.

#### Study Outcomes

The primary outcome was to identify distinct KAB profiles regarding the PHD among health care professionals. The secondary outcome was to investigate how sociodemographic characteristics relate to these profiles in this specific population.

### Statistical Analysis

#### Sample Size Calculation

On the basis of an estimated population of 8000 HCL staff members regularly using the hospital catering service (collective kitchen facilities), a minimum of 367 responses was calculated to ensure a 95% confidence level and a 5% margin of error for proportion estimates (finite population correction applied). This target was established to guarantee statistical precision for proportion estimates in the reference population.

#### Geospatial and Demographic Variables

Municipality-level variables were incorporated to explore contextual disparities, including population density; average annual income; and the Geographical Classification for Health Studies [30], which evaluates socioeconomic and environmental conditions.

#### Data Analysis

Descriptive statistics were used to analyze the sociodemographic and geographic characteristics of the participants and KAB scores.

To identify distinct profiles based on KAB dimensions, a hierarchical cluster analysis was first conducted using the Ward method and squared Euclidean distance. The optimal number of clusters was determined through visual inspection of the dendrogram. This structure was then used to guide the final k-means clustering procedure. A principal-component analysis was applied to visualize the separation of clusters.

To assess the association between individual characteristics and cluster membership, a multinomial logistic regression model was conducted. Independent variables included gender, age group, profession, dietary pattern, household size, population density, income quintile, Geographical Classification for Health Studies, and weekly food budget. Odds ratios (ORs) with 95% CIs and *P* values were reported.

In addition, a mediation analysis was conducted to examine whether attitudes mediated the relationship between knowledge and behavior. The analysis was based on the ordinary least squares regression method, and indirect effects were estimated via bootstrapping with 5000 samples. Unstandardized coefficients (*b*), SEs, 2-tailed *t* values, *P* values, and 95% CIs were reported for all effects. Statistical significance was set at *P*<.05.

### Ethical Considerations

This study was approved by the ethics committee of HCL (approval IRB00014232 2024 06 13_12, issued on June 19, 2024) and complied with the Declaration of Helsinki and the French data protection authority MR-004 norms. Written informed consent was obtained from all participants.

## Results

### Characteristics of the Participants

**Figure 1:**
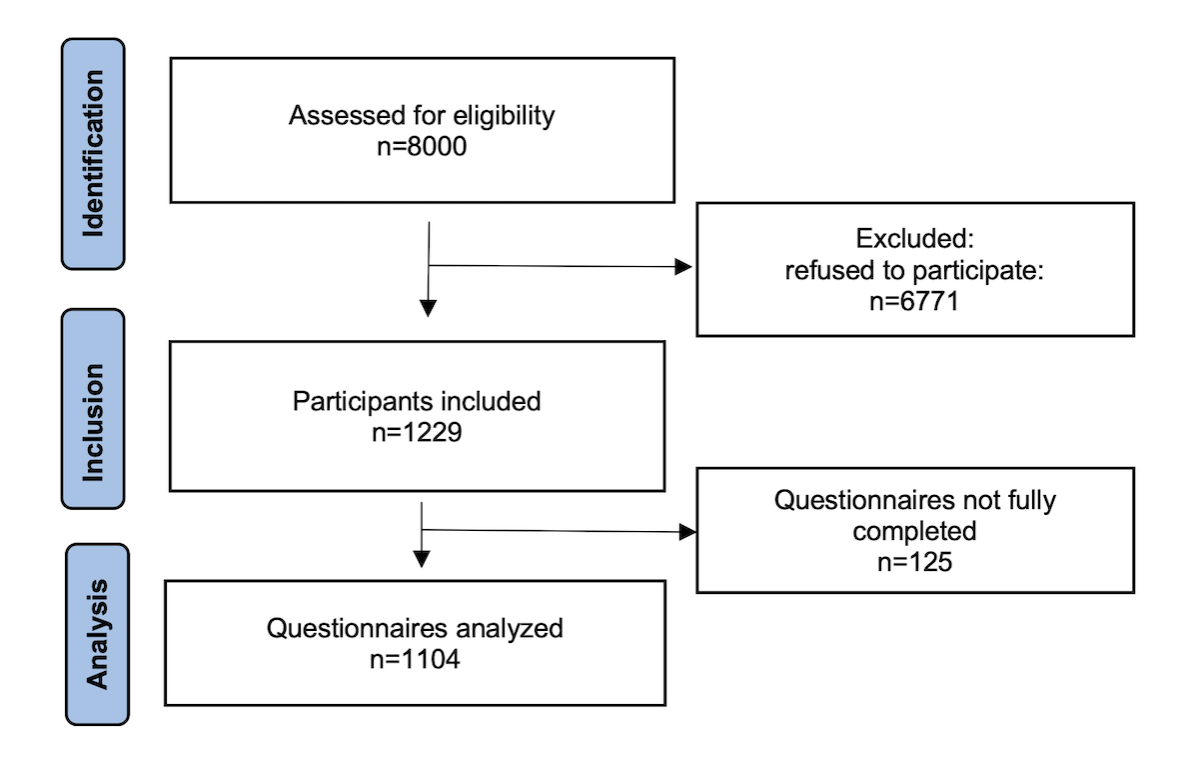
Flowchart of the study. Recruitment and inclusion process for a cross-sectional knowledge, attitudes, and behaviors survey on adherence to the planetary health diet among Hospices Civils de Lyon health care professionals (France; April 2024-June 2024).

Figure 1 shows the flowchart of the study. Of the 8000 persons assessed for eligibility, 1229 (15.36%) were included, and 1104 (13.8%) fully completed the questionnaire. This sample size, well above the calculated minimum, ensured high statistical precision (–2.7% to +2.7% margin of error), whereas the voluntary recruitment process may limit full population representativeness.

Table 1 shows the sociodemographic and geographic characteristics of the participants. Most participants were women (927/1104, 83.97%) aged between 30 and 59 years (882/1104, 79.89%), lived in small households (990/1104, 89.67%), and were in intermediate professions (720/1104, 65.22%). Omnivorous (702/1104, 63.59%) and flexitarian (331/1104, 29.98%) diets were predominant. Half of the participants (598/1104, 54.17%) lived in urbanized areas (quintile 4-5), and 69.02% (762/1104) resided in wealthy metropolitan areas. A high proportion (876/1104, 79.3%) belonged to municipalities in the top 2 income quintiles. Weekly food expenses varied, with 46.01% (508/1104) spending less than €100 (US $116.56) and 49.55% (547/1104) spending more.

**Table 1:** Sociodemographic and geographic characteristics of the study population and cluster membership (N=1104)^a^.

				All, n (%)		Cluster 1: knowledge-attitude gap (n=481), n (%)		Cluster 2: high intent, low action (n=318), n (%)		Cluster 3: behavior-driven alignment (n=305), n (%)
**Gender**										
	Women		927 (83.97)		389 (80.9)		270 (84.9)		268 (87.9)	
	Men		173 (15.67)		90 (18.7)		46 (14.5)		37 (12.1)	
	Other		4 (0.36)		2 (0.4)		2 (0.6)		0 (0)	
**Age group (y)**										
	18-29		146 (13.22)		68 (14.1)		30 (9.4)		48 (15.7)	
	30-39		263 (23.82)		112 (23.3)		86 (27)		65 (21.3)	
	40-49		295 (26.72)		138 (28.7)		74 (23.3)		83 (27.2)	
	50-59		324 (29.35)		143 (29.7)		95 (29.9)		86 (28.2)	
	≥60		76 (6.89)		138 (28.7)		134 (42.1)		65 (21.3)	
**Household size**										
	1-4 people		990 (89.67)		436 (90.6)		281 (88.4)		273 (89.5)	
	5-9 people		114 (10.33)		45 (9.4)		37 (11.6)		32 (10.5)	
**Profession**										
	Senior executive or intellectual employee		337 (30.53)		138 (28.7)		134 (42.1)		65 (21.3)	
	Intermediate profession		720 (65.22)		332 (69.0)		177 (55.7)		211 (69.2)	
	**Other categories**		47 (4.25)		11 (2.3)		7 (2.2)		29 (9.5)	
		Student	10 (0.91)		2 (0.4)		0 (0.0)		8 (2.6)	
		Laborer or worker	7 (0.63)		2 (0.4)		1 (0.3)		4 (1.3)	
		Other	30 (2.71)		7 (1.5)		6 (1.9)		17 (5.6)	
**Main diet**										
	Flexitarian		331 (29.98)		105 (21.8)		159 (50.0)		67 (22)	
	Omnivorous		702 (63.59)		362 (75.3)		118 (37.1)		222 (72.8)	
	**Other diets**		71 (6.43)		14 (2.9)		41 (12.9)		16 (5.2)	
		Pescatarian	13 (1.18)		2 (0.4)		8 (2.5)		3 (1.0)	
		Vegan	6 (0.54)		0 (0)		5 (1.6)		1 (0.3)	
		Vegetarian	30 (2.72)		4 (0.8)		22 (6.9)		4 (1.3)	
		Other	22 (1.99)		8 (1.7)		6 (1.9)		8 (2.6)	
**Population density** **categorized into** **quintiles** **(people per km^2^)**										
	Quintile 1		59 (5.34)		22 (4.6)		14 (4.4)		23 (7.5)	
	Quintile 2		171 (15.49)		68 (14.1)		46 (14.5)		57 (18.7)	
	Quintile 3		276 (25.00)		113 (23.5)		88 (27.7)		75 (24.6)	
	Quintile 4		205 (18.57)		90 (18.7)		64 (20.1)		51 (16.7)	
	Quintile 5		393 (35.60)		188 (39.1)		106 (33.3)		99 (32.5)	
**Income per consumption unit in quintiles per year**										
	Quintile 1 (€15,015-€18,072 [US $17,500.70-$21,063.80])		34 (3.08)		15 (3.1)		8 (2.5)		11 (3.6)	
	Quintile 2 (€18,110-€19,320 [US $21,108.10-$22,518.40])		99 (8.97)		31 (6.4)		37 (11.6)		31 (10.2)	
	Quintile 3 (€19,480-€20,732 [US $22,704.90-$24,164.20])		95 (8.60)		27 (5.6)		38 (11.9)		30 (9.8)	
	Quintile 4 (€20,774-€22,974 [US $24,213.10-$26,777.30])		430 (38.95)		208 (43.2)		114 (35.8)		108 (35.4)	
	Quintile 5 (€23,021-€36,562 [US $26,832.10-$42,614.80])		446 (40.40)		200 (41.6)		121 (38.1)		125 (41.0)	
**Geographical Classification for Health Studies**										
	Wealthy metropolitan areas		762 (69.02)		367 (76.3)		206 (64.8)		189 (62.0)	
	Precarious population districts		218 (19.75)		72 (15.0)		83 (26.1)		63 (20.7)	
	Periurban and rural areas		124 (11.23)		42 (8.7)		29 (9.1)		53 (17.4)	
**Weekly food budget**										
	€1-€50 (US $1.17-$58.28)		134 (12.14)		52 (10.8)		31 (9.7)		51 (16.7)	
	€51-€100 (US $59.44-$116.56)		374 (33.88)		172 (35.8)		109 (34.3)		93 (30.5)	
	€101-€150 (US $117.72-$174.83)		295 (26.72)		118 (24.5)		91 (28.6)		86 (28.2)	
	>€150 (US $174.83)		252 (22.83)		116 (24.1)		74 (23.3)		62 (20.3)	
	Did not know		49 (4.43)		23 (4.8)		13 (4.1)		13 (4.3)	

^a^Data from a cross-sectional survey on planetary health diet engagement conducted among health care professionals working at Hospices Civils de Lyon (France) between April 2024 and June 2024. Cluster allocation based on knowledge, attitudes, and behaviors profiles were derived from k-means analysis.

### Clustering of Health Care Professionals Based on KAB

**Figure 2:**
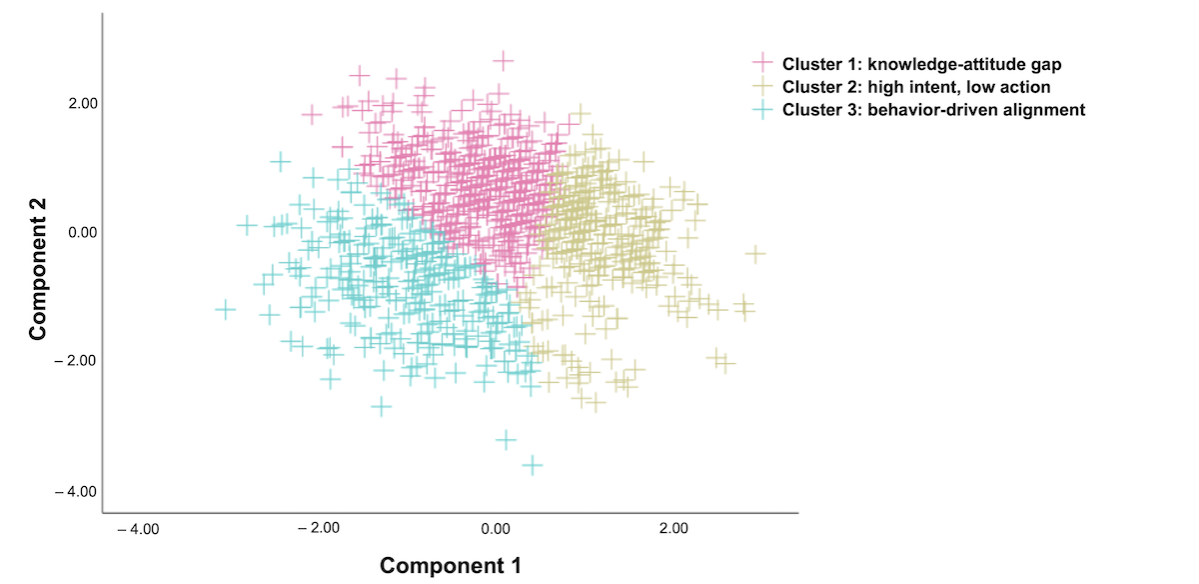
Principal-component analysis (PCA) of standardized knowledge, attitudes, and behaviors (KAB) scores related to the planetary health diet among health care professionals (N=1104). Each point represents an individual, colored by cluster membership from k-means analysis. The PCA revealed distinct KAB profiles.

To better understand variations in KAB toward the PHD, a cluster analysis was conducted using standardized KAB scores. The internal consistency of the solution was supported by a total within-cluster sum of squares of 103,797. This clustering allocation (Table 1) and analysis (Figure 2 and Table 2) revealed 3 distinct profiles in health care professionals’ engagement with the PHD.

Cluster 1, named “knowledge-attitude gap” (481/1104, 43.57%), exhibited a relatively high knowledge score (32.8) but moderate scores in both attitude (13.8) and behavior (14.6), resulting in a total KAB score of 61.2. Cluster 2, named “high intent, low action” (318/1104, 28.8%), had the highest knowledge (33.1) and attitude (23.3) scores but the lowest behavior score (10.2), yielding a total KAB score of 76.6. Cluster 3, named “behavior-driven alignment” (305/1104, 27.63%), had moderate knowledge (21.4) and attitude (11.7) scores and the highest behavior score (15.7), resulting in a total KAB score of 48.8.

**Table 2:** Descriptive analysis of knowledge, attitudes, and behaviors (KAB) scores by cluster^a^.

	Knowledge section score (0-40), mean (SD)	Attitude section score (0-40), mean (SD)	Behavior section score (0-40), mean (SD)	KAB score (0-120), mean (SD)
Cluster 1: knowledge-attitude gap (n=481)	32.8 (3.1)	13.8 (5.6)	14.6 (3.1)	61.2 (6.7)
Cluster 2: high intent, low action (n=318)	33.1 (4.4)	23.3 (4.8)	10.2 (3.4)	76.6 (6.0)
Cluster 3: behavior-driven alignment (n=305)	21.4 (3.6)	11.7 (6.3)	15.7 (3.7)	48.8 (8.2)

^a^Results from a cross-sectional survey on planetary health diet engagement conducted among health care professionals at Hospices Civils de Lyon, France (April 2024-June 2024). Clusters were identified through k-means clustering on standardized KAB scores to profile engagement patterns with the PHD.

### Sociodemographic, Geographic, and Clinical Characteristics of the Clusters

Cluster 1 (“knowledge-attitude gap”; 481/1104, 43.57% of the participants) was predominantly composed of middle-aged women in intermediate professions with a largely omnivorous diet and living in wealthy metropolitan areas (Table 1). Cluster 2 (“high intent, low action”; 318/1104, 28.8% of the participants) included more older participants and had the highest proportion of flexitarians. This group showed more diversity in occupational status and place of residence, with more frequent location in low- and middle-income municipalities. Cluster 3 (“behavior-driven alignment”; 305/1104, 27.63% of the participants) was the youngest group, also predominantly female, with mixed diets and a more frequent location in rural areas. Weekly food expenses were similar across clusters, mostly ranging from €51 to €150 (US $59.44-$174.83).

### Identification of Sociodemographic, Geographic, and Clinical Determinants of Low Engagement and Cluster Alignment Toward the PHD

**Figure 3:**
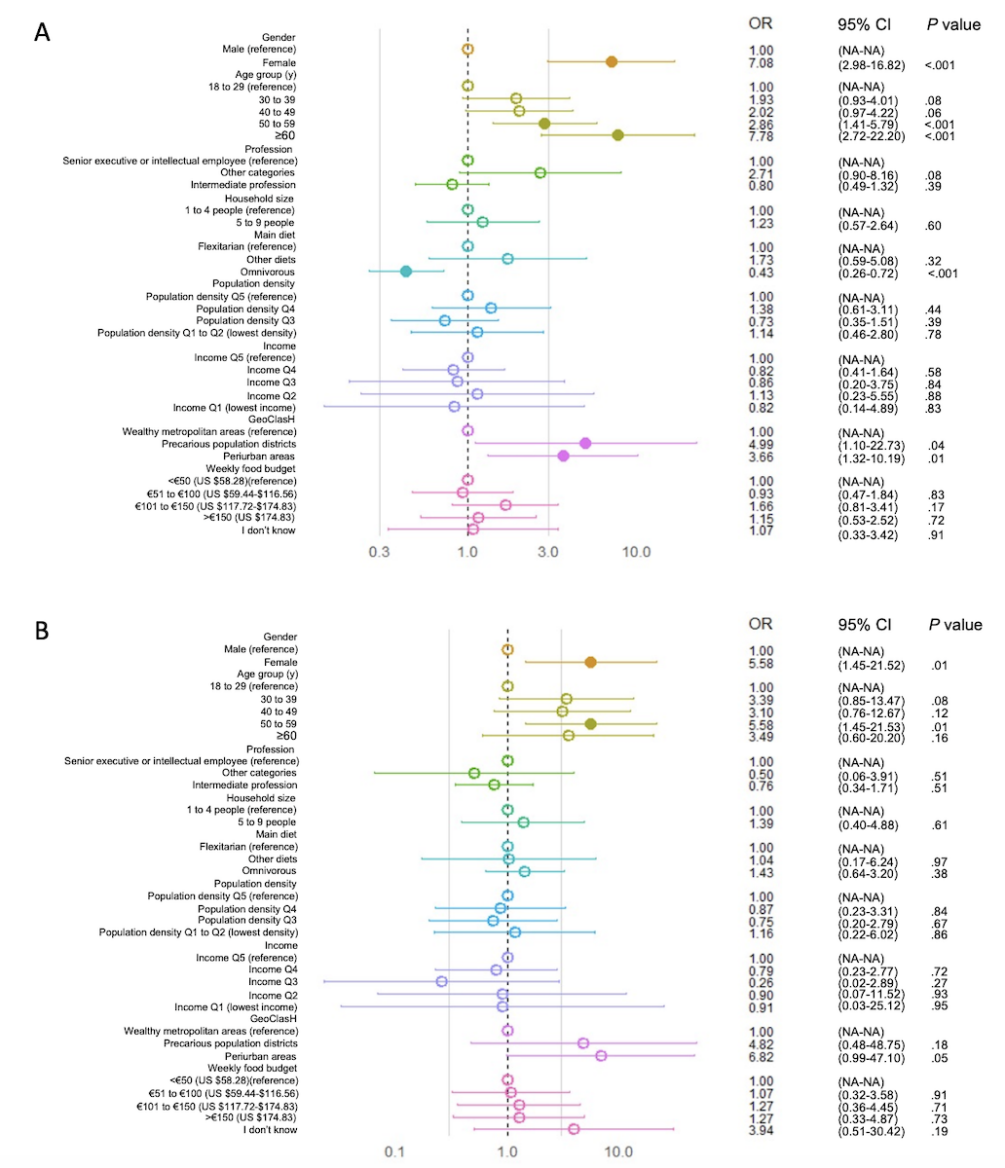
Sociodemographic and geographic determinants of low engagement and alignment clusters with the planetary health diet. (A) Comparison of cluster 1, “knowledge-attitude gap,” with cluster 3, “behavior-driven alignment.” (B) Comparison of cluster 2, “high intent, low action,” with cluster 3, “behavior-driven alignment.” An empty circle indicates a *P* value greater than .05, whereas a filled circle indicates a *P* value of .05 or less. GeoClasH: Geographical Classification for Health Studies; NA: not applicable; OR: odds ratio; Q: quintile.

A multinomial logistic regression evaluated associations between individual characteristics and cluster membership considering the “behavior-driven alignment” cluster (cluster 3) as the reference category (Figure 3).

The comparison of cluster 1, “knowledge-attitude gap,” with cluster 3, “behavior-driven alignment” (Figure 3A), demonstrated that cluster 1 was significantly associated with being female (OR 7.08, 95% CI 2.98-16.8; *P*<.001) and of older age, especially ≥60 years (OR 7.78, 95% CI 2.72-22.20; *P*<.001) and 50 to 59 years (OR 2.86, 95% CI 1.41-5.79; *P*=.004). Omnivorous individuals were less likely than flexitarians to belong to cluster 1 (OR 0.43, 95% CI 0.26-0.72; *P*=.001). Living in periurban areas (OR 3.66, 95% CI 1.32-10.19; *P*=.01) or precarious population districts (OR 4.99, 95% CI 1.10-22.73; *P*=.04) also increased the probability of alignment with this cluster. Other variables showed no significant associations.

The comparison of cluster 2, “high intent, low action,” with cluster 3, “behavior-driven alignment” (Figure 3B), demonstrated that the cluster 2 profile was more likely associated with women (OR 5.58, 95% CI 1.45-21.52; *P*=.01) and individuals aged 50 to 59 years (OR 5.58, 95% CI 1.45-21.53; *P*=.01). A marginal trend of alignment with this cluster was noted for those in periurban areas (OR 6.82, 95% CI 0.99-47.10; *P*=.05). No other factors were significantly associated.

### Exploration of Mediating KAB Relationships

The results of the mediation analysis (Table 3) revealed that knowledge significantly predicted attitudes (*b*=0.2893 (0.2230-0.3556); SE 0.0338; t_1102_=8.56; *P*<.001). In turn, attitudes significantly predicted behaviors (*b*=0.1798, 95% CI 0.1472-0.2124; SE 0.0166; t_1102_=10.83; *P*<.001). However, the direct effect of knowledge on behaviors controlling for attitudes was not statistically significant (*b*=0.0072, 95% CI −0.0306 to 0.0449; SE 0.0192; t_1102_=0.37; *P*=.71).

The indirect effect of knowledge on behaviors via attitudes was significant (*b*=0.0520), with a 95% CI (0.0379-0.0682) that did not include 0. This indicates a full mediation effect: knowledge influences behaviors only indirectly through its effect on attitudes.

**Table 3:** Mediation analysis examining the indirect effect of attitudes (A) in the relationship between knowledge (K) and behavior (B) (K→A→B)^a^.

Path	Coefficient (*b*; SE; 95% CI)	*t* test	*P* value
Effect of knowledge on attitude (path a)	0.2893 (0.0338; 0.2230 to 0.3556)	8.56	<.001
Effect of attitude on behavior (path b)	0.1798 (0.0166; 0.1472 to 0.2124)	10.83	<.001
Direct effect of knowledge on behavior controlling for attitude	0.0072 (0.0192; −0.0306 to 0.0449)	0.37	.71
a × b (indirect effect)^b^	0.0520 (0.0077; 0.0379 to 0.0682)	—^c^	—

^a^Data from a cross-sectional knowledge, attitudes, and behaviors survey on planetary health diet adherence among health care professionals working at Hospices Civils de Lyon (France; April 2024-June 2024).

^b^Estimated via bootstrapping (5000 samples).

^c^Empty cells indicate that t or *P* values were not computed for the indirect effect.

## Discussion

### Principal Findings and Comparison With Prior Work

In a French context and among HCL health care professionals, this study addressed global health by identifying key behavioral typologies regarding the PHD. The PHD represents a transformative framework for addressing interlinked crises of human health and environmental sustainability. However, our findings highlight persistent gaps in its adoption even among health-competent populations. Cluster analysis of KAB identified three distinct profiles: (1) “knowledge-attitude gap”; (2) “high intent, low action”; and (3) “behavior-driven alignment.” These profiles underline both opportunities and barriers shaping dietary transitions within health care systems.

The largest cluster, “knowledge-attitude gap” (481/1104, 43.57% of the participants) demonstrated high knowledge about the PHD but failed to adopt corresponding dietary behaviors. This knowledge-behavior disconnect has been noted across public health domains, particularly among health care workers [31,32], who have dual responsibilities: modeling health-promoting behaviors and guiding patients toward sustainable lifestyles. Similar gaps have been identified in tobacco cessation [33] and preventive oncology [34], suggesting that professional knowledge does not ensure personal action. Our analysis is consistent with the KAB framework, indicating that, within this sample, knowledge appeared to influence behaviors indirectly through attitudes. Thus, affective and motivational factors may be as critical as informational ones. Environmental factors, institutional norms, and perceived behavioral control likely moderate this pathway, highlighting the importance of structural interventions alongside education [19,35]. Our mediation analysis suggests that targeted interventions should emphasize strengthening attitudes and motivations rather than simply providing information. Approaches such as motivational interviewing, normative messaging, and role-modeling within health care teams might significantly reduce the knowledge-attitude gap [36,37].

The “high intent, low action” cluster (318/1104, 28.8% of the participants) demonstrated high knowledge and positive attitudes but low PHD-aligned behaviors. This profile illustrates a different form of behavioral inertia: intention without implementation. Contributing factors may include workplace culture, lack of time, workplace food options, or insufficient social support. Previous studies show that, without standardized and accessible sustainable consumption options, even health-conscious individuals struggle to act on their intentions [38]. This profile was more prevalent among older and flexitarian participants, suggesting generational or cultural barriers to adopt the PHD. These individuals may benefit from interventions promoting action planning and institutional support, such as providing plant-based meals in hospitals or developing peer-led sustainability programs.

Conversely, the “behavior-driven alignment” cluster (305/1104, 27.63% of the participants), characterized by the highest behavioral alignment, had only moderate knowledge and attitude scores. This challenges the assumption that knowledge is a prerequisite for sustainable dietary practice. It underscores the role of social and contextual determinants on food choices. Behavioral science research suggests that daily routines, cultural norms, and environmental prompts can foster health-promoting behaviors even without strong cognitive drivers [39,40]. This cluster, predominantly young and periurban, may reflect evolving dietary behaviors driven by cultural trends, peer influence, or institutional exposure rather than formal health education [41,42]. Exploiting these archetypes could inform peer-led interventions within health systems.

Collectively, these profiles reveal that no group simultaneously achieved high knowledge and high behavioral adherence to the PHD. This overall pattern underscores the persistence of a structural knowledge-behavior gap across all segments. The absence of a cluster combining both high knowledge and high adoption of PHD practices reflects the well-established knowledge-behavior gap in health psychology and sustainability research. Numerous studies have shown that knowledge or intention alone seldom predicts actual behavior as the translation into action depends on psychological, social, and contextual moderators [43,44]. Self-efficacy and perceived behavioral control play pivotal roles in bridging this gap, influencing whether individuals feel capable of acting upon what they know [45]. Despite the proliferation of evidence-based guidelines, barriers to adherence persist (eg, guideline complexity, environmental context, and resource constraints)—as documented in recent implementation research [46,47]. Frameworks highlight multistage processes in which motivation, environmental triggers, and sustained self-regulation determine whether intentions lead to consistent behavioral adoption [48]. Similar intention-action discrepancies have been observed in sustainable food consumption, where positive attitudes coexist with low behavioral uptake due to contextual and social constraints [49]. Altogether, these findings suggest that promoting sustainable dietary practices among health care staff requires multilevel interventions that combine education with environmental and organizational enablers.

Beyond knowledge and attitudes, professional identity and perceived institutional norms significantly influence behavior. When sustainability is not embedded in the clinical culture, a disconnect can arise between personal values and visible action. Positioning the PHD as a personal choice and a professional standard could reinforce adoption and enable culture change [50]. Interventions integrating social role-modeling, normative messaging, and organizational alignment with PHD principles may be more effective than education alone.

The coexistence of these 3 profiles within a single health system reveals that health care professionals, despite having received scientific training and facing NCDs, do not uniformly model sustainable dietary behaviors. Lifestyle behaviors among health care professionals reflect inconsistencies found in the general population [51]. Given their direct involvement in managing NCDs and their role as trusted communicators, health care professionals’ dietary practices influence public health far beyond personal well-being [52]. By adopting and advocating for sustainable diets aligned with frameworks such as the PHD, which integrates human and environmental health goals, they can enhance clinical credibility and support institutional shifts toward healthier food policies [53,54].

Both the WHO [18] and the EAT-Lancet Commission [1] emphasize the PHD potential to reduce premature mortality, alleviate environmental degradation, and contribute to the United Nations Sustainable Development Goals [55]. Therefore, aligning health care professional behavior with these recommendations is a systemic opportunity. A PHD-adherent workforce is not only healthier but also more credible in promoting planetary health.

Health care institutions must recognize that individual knowledge is not sufficient to drive change. Behavioral interventions need embedding within broader institutional commitments to sustainability, including food supply policies, cafeteria reforms, cooking staff education, and food environment restructuring. Differentiating between structural barriers (eg, food access) and psychological barriers (eg, habit and motivation) should enable targeted actions on both fronts. Systems combining policy shifts with environmental engagements such as menu redesign, sustainable meal defaults, or workplace programs are more likely to succeed [56]. By integrating PHD principles into daily operations and wellness programs, health systems can act as exemplars and catalysts for the planetary health agenda, contributing directly to climate resilience, disease prevention, and institutional leadership [57].

### Limitations

This study has several limitations. First, it was conducted in a single hospital network and among health care professionals only using a convenience sampling strategy. These features may limit external validity and prevent direct extrapolation to the general population or other institutional contexts. In addition, analyses were descriptive by design, intended to identify behavioral profiles rather than develop predictive models. Second, self-reported data may have introduced social desirability bias, particularly among health care professionals who may overreport sustainable behaviors. Third, the cross-sectional design limits the ability to infer causality or examine the progression from knowledge to behavior over time, highlighting the need for longitudinal studies. Fourth, the sample was disproportionately female, reflecting HCL staffing demographics (75% women) but potentially influencing the results and limiting representativeness. Fifth, the study did not distinguish between clinical staff directly involved in patient care and administrative personnel. In addition, variables such as seniority or cultural or ethnic background were not collected due to institutional and regulatory restrictions, which may have constrained the exploration of factors influencing KAB. Sixth, although structural and institutional influences were conceptually discussed, they were not directly measured, limiting empirical assessment of their moderating effects. Finally, while the PHD offers a globally relevant framework, its applicability varies across cultural and socioeconomic contexts, requiring local adaptation. Future research should explore institutional culture, perceived norms, and structural facilitators to better understand their specific roles in shaping dietary adoption.

### Perspectives

In French hospital settings (like the HCL), the 3 KAB profiles can guide targeted action: improve motivation and perceived control for the knowledge-attitude gap; remove practical barriers and adjust the food environment for high intent, low action; and maintain momentum for behavior-driven alignment. Indeed, given its scale and structure, HCL provides a relevant pilot environment to test and refine such approaches before broader implementation across other health care systems. As one of the largest public hospital networks in France [23], it offers a realistic institutional platform to evaluate feasibility and inform national deployment strategies.

Short-term institutional priorities are to align cafeteria offerings with the PHD (default options, placement, and pricing), deliver brief training with clear normative cues, and track change over time using the current KAB scoring framework (light-touch monitoring).

Medium-term priorities are to replicate the approach in other French health care contexts and link results to routine indicators (diet adherence, purchasing, and waste) to evaluate health and sustainability impacts. If routine standardized measurement is needed, a separate program could undertake formal psychometric validation; this is not required to implement the aforementioned actions.

### Conclusions

Our study reveals heterogeneity in how health care professionals relate to the PHD. While knowledge remains important, attitudes and contextual factors strongly shape behavior. The identification of a behaviorally aligned cluster with only moderate knowledge underlines the power of cultural and environmental levers. To advance planetary health, health care must move beyond education toward institutional alignment. Within hospital systems, health care professionals may play an important role in promoting alignment with planetary health principles, potentially contributing to both personal and institutional sustainability goals.

Practical steps such as policy reform, sustainable food guidelines, staff training, and integrating sustainability into curricula are essential to embed these values within health care systems and support meaningful, long-term dietary transformation.

## Appendix

Multimedia Appendix 1 Checklist for Reporting of Survey Studies (CROSS).

## Abbreviations

HCL: Hospices Civils de Lyon
KAB: knowledge, attitudes, and behaviors
NCD: noncommunicable disease
OR: odds ratio
PHD: planetary health diet
WHO: World Health Organization

## Data Availability

The datasets generated or analyzed during this study are available from the corresponding author on reasonable request.
